# Supplementary material for: Complexome profiling of the Chlamydomonas psb28 mutant reveals TEF5 as an early PSII assembly factor
Source: Plant Cell. 2025 Mar 17;37(6):koaf055. doi: 10.1093/plcell/koaf055 (PMC12164590; doi:10.1093/plcell/koaf055)
Supplement: koaf055_Supplementary_Data [file koaf055_supplementary_data.zip › psb28_interactive.zip!/plots/intensity/qualityPlots/peptides/NUOB8_Cre16.g679500.t1.2.html]

### Cre16.g679500.t1.2

Trivial name: NUOB8  
SeqCoverage [median]: 65.30  
Mass: 11.00 kDa  
  
 

| Sequence | MissedCleavage | Mass [Da] | wtIntensity | muIntensity | Score |
| --- | --- | --- | --- | --- | --- |
| ANPHFPILIR | 0 | 1176.68 | 5045.77 | 706.82 | 90.15 |
| DFIVANYAEMK | 0 | 1299.62 | 3253.25 | 0.00 | 80.69 |
| DFIVANYAEMKK | 1 | 1427.71 | 765.47 | 1086.97 | 74.16 |
| GVRDFIVANYAEMKK | 2 | 1739.90 | 0.00 | 1115.69 | 46.93 |
| SMQELR | 0 | 762.37 | 4050.67 | 1763.71 | 132.09 |
| SVNVQGATTGAVQQQLEQLILAGQSMPK | 0 | 2895.51 | 4435.21 | 0.00 | 112.80 |
| YDFGAEK | 0 | 828.37 | 3334.51 | 6342.41 | 114.78 |
|  |  |  | 20884.9 | 11015.6 |  |
